# Disconnection syndromes and injury to neural systems after ischemic stroke

**DOI:** 10.3389/fstro.2025.1643570

**Published:** 2026-01-23

**Authors:** Anne Schwarz, Christina K. Holl, Lorie Brinkman, Andrea J. Stehman, Isabel Cardoso Ferreira, Nina Soleimani, Eden Farahmand, Maeve Settle, Shivani Sakthi, Ivy Vo, Natalie Olivares, Min-Keun Song, Steven C. Cramer

**Affiliations:** 1 Department of Neurology, David Geffen School of Medicine at UCLA, Los Angeles, CA, United States; 2 California Rehabilitation Institute, Los Angeles, CA, United States; 3 Division of Biokinesiology and Physical Therapy, University of Southern California, Los Angeles, CA, United States; 4 Department of Physical & Rehabilitation Medicine, Chonnam National University Medical School, Gwangju, Republic of Korea

**Keywords:** disconnection, imaging biomarkers, motor symptoms, neural injury, neural networks, non-motor symptoms, stroke

## Abstract

**Background:**

Stroke-related impairments present in wide-ranging combinations, including cognitive and upper extremity (UE) sensorimotor deficits, complicating an understanding of their relationship with the anatomy of injury. Here, we hypothesized that deficits in UE sensorimotor function, mood, and cognition would be associated with distinct patterns of neural injury, and we explored whether complex outcome measures that make both cognitive and motor demands are more vulnerable to injury-related disconnection after stroke.

**Methods:**

Subject testing included elementary sensorimotor behaviors (shoulder and finger strength [SAFE], Fugl-Meyer Assessment [FMUE], and wrist proprioception [WPST]), complex behaviors that require substantial motor and cognitive control (Box and Blocks Test [BBT] and Trail Making Test-A [TMT-A]), cognition (Montreal Cognitive Assessment [MoCA]), and mood (Geriatric Depression Scale). Infarcts were outlined on clinical scans and used to compute lesion volume, injury to the corticospinal tract (CST) as well as thalamocortical sensory tract, and measures of structural network disconnection. Associations between lesions and behavior were examined using three methods: [1] voxel-lesion-symptom mapping (VLSM) to identify lesioned voxels associated with behavioral deficits; [2] correlation, to identify bivariate relationships between neuroimaging and behavioral measures; and [3] LASSO regression to identify the most behaviorally relevant variables among neuroimaging and clinical measures.

**Results:**

Stroke patients (*n* = 55, mean age 69.2, 42% females) had lesion volumes ranging from 0.1 to 354.9 (mean 30.9) ml and averaged 10.4 ± 4.9 days post-stroke. Deficits in all three elementary UE sensorimotor behaviors (SAFE, WPST, FMUE) correlated with extent of injury to CST not disconnection measures, with VLSM largely consistent, while deficits in complex motor and cognitive behaviors (BBT and TMT-A) were related to widespread structural disconnection between brain networks. LASSO models that consider all neuroimaging and clinical measures revealed complex patterns of disconnections across behaviors.

**Conclusion:**

These findings indicate that elementary UE sensorimotor behaviors are related to the integrity of regional sensorimotor system structures, but that more complex motor and cognitive behaviors are more related to intact structural connectivity between multiple brain networks.

## 1 Introduction

Stroke remains a leading cause of human disability (Feigin et al., 2025). Stroke-related deficits commonly include sensorimotor and non-motor cognitive impairments, with varying severity, and overlapping syndromes (Gittins et al., 2021; Mullick et al., 2015). Upper extremity (UE) motor impairments were reported to occur in 75.5% (Rathore et al., 2002), and cognitive impairments in 60% of stroke survivors (El Husseini et al., 2023). Such deficits have been linked to damage in specific brain regions (Bisogno et al., 2021).

Neuroimaging techniques have linked stroke-related motor impairments to injury in several brain regions. For example, the motor system is described as having “elegant” organization, and motor deficits are related to the integrity of specific structures such as the corticospinal tract (CST). Measures of injury to the CST are related to hand impairment, and its recovery and have been recommended for use as a biomarker in clinical trials (Lin et al., 2019; Boyd et al., 2017). Furthermore, it was recently established that structural integrity of the CST had the highest accuracy in terms of sensitivity and specificity for discriminating between stroke subjects with and without arm weakness, showing an AUC of 0.9 (Yoo et al., 2023). Similarly, finger proprioception, (Ingemanson et al., 2019a), and its improvement with therapy, (Ingemanson et al., 2019b) have been related to sensory system injury using a measure that includes damage to the thalamocortical sensory tract (TST).

More complex motor tasks, such as those that require sustained attention, also rely on additional cognitive processes, including motor planning, initiation, attention, and working memory (Roby-Brami et al., 2021; Mullick et al., 2015). These cognitive processes rely on interactions with additional brain networks, and so deficit severity for motor tasks with higher cognitive demand may be expected to correlate with degree of injury to multiple neural systems, motor and non-motor. Indeed, several studies have reported on the overlap between motor and cognitive functions after stroke (Gittins et al., 2021; Einstad et al., 2021; Lin et al., 2021). Neuroanatomical correlates of cognitive impairments, such as attentional deficits, involve large-scale multimodal attention networks distinct from sensorimotor networks (Murakami et al., 2014). Few studies have examined multiple behaviors and neural systems in parallel, though such an approach may be useful to understand the basis for post-stroke deficits in cognitively demanding sensorimotor tasks.

One prior study found distinct sites of brain injury when comparing grip strength, an elementary task low in attentional demand, to the Box and Blocks Test (BBT), a complex task high in attentional demand. Grip strength was associated with injury to sensorimotor structures, such as the peri—Rolandic cortex and CST, whereas BBT performance was associated with injury to more diffuse brain regions beyond the sensorimotor cortex, including orbitofrontal cortex and the dorsal anterior insula, a region known to be important for complex executive function (Lin et al., 2021).

Together, these studies suggest that deficits in elementary behaviors are related to injury to a restricted set of regional sensorimotor brain structures, while deficits in more complex behaviors that require motor and cognitive activity are related to more diffuse forms of brain injury that cause structural disconnection between networks. The current study therefore hypothesized that impairments in elementary UE sensorimotor tasks are correlated with injury to specific brain sensorimotor structures such as the CST, while deficits in complex motor and cognitive tasks are additionally related to structural disconnection between brain networks.

## 2 Materials and methods

Adults with stroke onset in the past 30 days were recruited during admission to a single inpatient rehabilitation facility. Exclusion criteria included a major active disease that affected UE function (apart from the index stroke) or deficits in cognition or communication that would interfere with study procedures. All subjects signed informed consent, and the study was planned in adherence with the Declaration of Helsinki and approved by the University of California, Los Angeles Institutional Review Board (IRB#21-000936). Age, sex, days after stroke, and the paretic side were collected for each study participant as baseline demographics. All outcomes in this study were restricted to baseline assessments to allow a cross-sectional analysis with a minimal temporal gap between impairment measures and neuroimaging data.

### 2.1 UE motor and cognitive outcome measures

Elementary sensorimotor deficits in the affected UE were measured via tests of proximal and distal arm strength wrist proprioception UE motor impairment and gross manual dexterity. Strength was measured using the shoulder abduction, and finger extension (SAFE) score, which rates each of these two movements in accordance with the Medical Research Council (MRC) Scale for Muscle Strength from 0 (no muscle activation) to 5 (activation against full resistance). Proprioception sense of the contralesional wrist was defined as the average error of the wrist flexion-extension angle over 20 consecutive trials at predetermined angles using the wrist proprioception sense test (WPST) apparatus (Carey et al., 1996; Young et al., 2022). UE motor impairment was measured using the motor Fugl-Meyer Assessment of the Upper Extremity (FMUE) (See et al., 2013; Fugl-Meyer et al., 1975), which has 33 test items that span dimensions such as strength, range of motion, reflexes, and selective joint control, with a maximum total score of 66 points.

Two tests that admix sensorimotor behavior with a complex combination of cognitive functions were also scored. Unilateral gross manual dexterity was measured using the Box and Blocks Test (BBT) that assesses how many 1 inch^3^ wooden blocks a person can move from one side of the box to the other, across a 15 cm-high barrier, within 60 s (Mathiowetz et al., 1985). The BBT performance is closely related to activities of daily living performed with the UE in person after stroke (Kim and Shin, 2022) and is explained by up to 33% percent by cognitive deficits (Lin et al., 2021). The Trail Making Test-A (TMT-A) was assessed as it is commonly used in neuropsychological assessment batteries in stroke rehabilitation and research (Tsiakiri et al., 2024). This test captures several cognitive behaviors, including executive function, attentional shift, psychomotor speed, abstraction, flexibility, numeric sequencing, and visual scanning while subjects perform a sensorimotor task connecting 25 numbers on a sheet of paper (Salthouse, 2011). Both, the BBT, and TMT-A, are more complex than the elementary sensorimotor behavioral tests, because they rely on maximum performance and multiple motor and cognitive skills (e.g., psychomotor speed, sustained attention) under time-constrained conditions, when executing iterative grasping, and release movements in the BBT or drawing multiple continuous lines between numbers in the TMT-A, while memorizing a set of rules (e.g., only one block at a time, no throwing, do not skip numbers, do not lift pencil from the paper).

Cognitive dysfunction was screened using the Montreal Cognitive Assessment (MoCA), which has been shown to be predictive of poststroke cognitive impairments (Chiti and Pantoni, 2014). The MoCA contains seven domains: visuospatial/executive, naming, attention, language, abstraction, memory, and orientation; these are summed to a maximum possible score of 30 points (Nasreddine et al., 2005).

Depression symptoms were measured using the 15 question version of the Geriatric Depression Scale (GDS), which has favorable clinimetric properties in stroke patients during the rehabilitation phase and has been validated for use in a broad range of the stroke population (Sivrioglu et al., 2009; Agrell and Dehlin, 1989; Roger and Johnson-Greene, 2009).

### 2.2 Lesion segmentation

Ischemic lesions were outlined on diffusion-weighted images (DWI) acquired during the acute stroke admission, informed by fluid-attenuated inversion recovery (FLAIR) and T1-weighted images when available. Lesion masks were manually segmented using Insight Segmentation and Registration Toolkit—Simple Not Another Picture Archiving, and Communication System (ITK-SNAP) (Yushkevich et al., 2006) by trained team members who were blinded to clinical data except for the lesion side and supervised by an experienced neuroimaging researcher (AS). The training was based on a manual with instructions on using the neuroimaging segmentation software ITK-SNAP and a brief guidance in stroke neuroanatomy and lesion identification as described by Lo et al. (2022). The authors report excellent inter-rater reliability (intraclass correlation 0.98) and Dice similarity coefficients of 0.7 (Lo et al., 2022). All lesion masks were reviewed and approved by a vascular neurologist experienced in neuroimaging research (SCC). The native-space lesion masks were then normalized to standard stereotaxic Montreal Neurologic Institute (MNI) space using a processing pipeline that included a high-definition brain extraction tool (HD-BET) (Isensee et al., 2019) for skull stripping of T1-weighted images along with Advanced Normalization Tools (ANTS) (Avants et al., 2008) for both rigid and non-linear transformation of DWI scans. All normalized and binarized masks were visually reviewed and approved and were then used to run the voxel lesion symptom mapping and to calculate lesion volume regional injury measures and structural network disconnections.

### 2.3 Lesion-related imaging measures

#### 2.3.1 Measures of regional injury

Lesion volume, in ml, was calculated based on the normalized lesion mask in MNI space.

The extent of injury to three different CSTs, originating in either the primary motor cortex (M1), dorsal premotor cortex (PMd), or ventral premotor cortex (PMv), was calculated based on the percentage of the tract's 16 longitudinal subsections at a 5% injury overlap threshold (Riley et al., 2011; Cassidy et al., 2018; Lin et al., 2019).

The extent of injury to the TST was calculated in a similar way as the percentage of its subsections at a 5% injury overlap threshold (Ingemanson et al., 2019a,b).

#### 2.3.2 Measures of structural network disconnection

Structural network connectivity was evaluated by using the NeMo network modification tool, which estimates the effects of an injury mask on white matter integrity, or structural connectome connectivity (Kuceyeski et al., 2013). Each binary lesion mask was superimposed onto a large tractography reference dataset comprising 420 individuals from the Human Connectome Project's (HCP) 1200 release (50 percent female, aged 25–35) to estimate structural disconnections (Olafson et al., 2021). The estimated structural connectivity is expressed as a change in connectivity (ChaCo) score based on the percent of damaged white matter streamlines at each voxel and ranges from 0 for no disconnection to 1 for complete disconnection (Kuceyeski et al., 2014). For this study, the binarized and normalized lesion masks were processed using the NeMo cloud interface (NeMo v2.1.3 – 2025-01-27 [github docs], online accessed on 2025-03-28, https://kuceyeski-wcm-web.s3.amazonaws.com/nemo/index.html). In order to quantify effects on the network level, the pairwise ChaCo scores were further parceled according to the seven-network cortical parcellation by Yeo and colleagues (Yeo et al., 2011). This network parcellation was derived from resting-state fMRI data of 1,000 subjects and includes the visual (VN), somatomotor (SMN), dorsal attention (DAN), ventral attention (VAN), limbic (LMN), frontoparietal control (FPN), and default mode (DMN) networks (Yeo et al., 2011). These networks were reported to be involved in visual processing (VN), sensorimotor functions (SMN), directing attention, and visuospatial processing (DAN), salience detection, and changing the attentional focus (VAN), emotional, and memory processing (LMN), working memory, and task control (FPN), and internally focused thought processing during rest (DMN) (Ishida et al., 2023).

### 2.4 Voxel lesion symptom mapping

Voxel-based lesion symptom mapping (VLSM) was performed to define regions of the brain where injury is associated with greater severity of specific post-stroke deficits, specifically with elementary sensorimotor behaviors (SAFE, WPST, and FMUE), complex motor and cognitive behaviors (BBT, and TMT-A) and cognitive (MoCA) and mood (GDS) deficits. Using the MNI space lesion masks, left hemispheric lesions were flipped to the right hemisphere and analyzed with NiiStat for Matlab (http://www.nitrc.org/projects/niistat). VLSM was performed on voxels that were injured in at least 5% of the subjects, using a voxel-based permutation threshold of 3,000 permutations for multiple comparison correction, and correction for the autocorrelation structure of lesioned voxels (Winkler et al., 2014).

### 2.5 Statistical analysis

The data were presented as mean and standard deviation, median and interquartile range, or frequencies depending on the variable characteristics. All analyses were performed using R 4.3.2 (R Foundation for Statistical Computing 2021 Vienna Austria).

In contrast to the VLSM that assesses the direct effects of the injury on behavior by means of lesioned voxels, the indirect effects of the lesion on relevant brain regions (e.g., CST or TST) and structural network connectivity (e.g., structural disconnections across the seven brain networks) and their association with the different behavioral deficits was further tested in correlation and regression analysis. Spearman rank correlation coefficients were computed to determine the relationship that each behavioral outcome (three elementary UE sensorimotor measures [SAFE, WPST, and FMUE] two complex motor and cognitive behaviors [BBT, and TMT-A], cognition [MoCA], and mood [GDS]) had with lesion-related neuroimaging measures (lesion volume, four regional tract injury measures [M1CST, PMv tract, PMd tract, and TST] and 28 structural network disconnections between the seven large-scale networks [VN, SMN, DAN, VAN, LMN, FPN, and DMN]); these results are presented with and without false discovery rate (FDR) correction to control for the proportion of false positives among significant results in multiple comparisons (Lindquist and Mejia, 2015). FDR correction is widely used in neuroscience and genetics and especially recommended for spatially correlated neuroimaging data. FDR corrections provide a better balance between controlling Type I and Type II errors when compared to more conservative approaches such as Bonferroni that eliminate the false positive Type I error but increase the Type II error or the statistical power needed. Significant correlations between disconnections among the seven neural networks, and behavioral outcomes were illustrated using chord diagrams. The strength of the correlation coefficients was interpreted as negligible if less than *r* = 0.1, weak if less than *r* = 0.4, as moderate between *r* = 0.4 and 0.69, as strong if between *r* = 0.7 and 0.89, and as very strong if *r* = 0.90 or higher (Schober et al., 2018).

In addition, multivariable regression analysis was used to explore the effects of the different neuroimaging measures for explaining the variance in baseline behavioral deficits (SAFE, WPST, FMUE, BBT, TMT-A, MoCA, and GDS) as dependent variables in separate models. The full set of independent candidate variables for each model includes the 33 neuroimaging measures (lesion volume, 4 regional tract injury measures, 28 structural network disconnection measures) and 4 clinical covariates (age, sex, paretic side, and days after stroke). Since 37 candidate variables would be highly susceptible to model overfitting and unstable coefficient estimates given the sample size, each regression model employed least absolute shrinkage and selection operator (LASSO) to identify the most relevant candidate variables. LASSO is a regularization technique that applies a penalty on the absolute size of the regression coefficient by shrinking the less important to zero while controlling for potential multicollinearity among variables (Tibshirani, 1996). Each model's performance was evaluated using R^2^ and R^2^ obtained under 4-fold cross-validation repeated 10 times to assess model robustness, and to provide an internal form of external validation in the absence of an independent dataset.

## 3 Results

**Figure 1:**
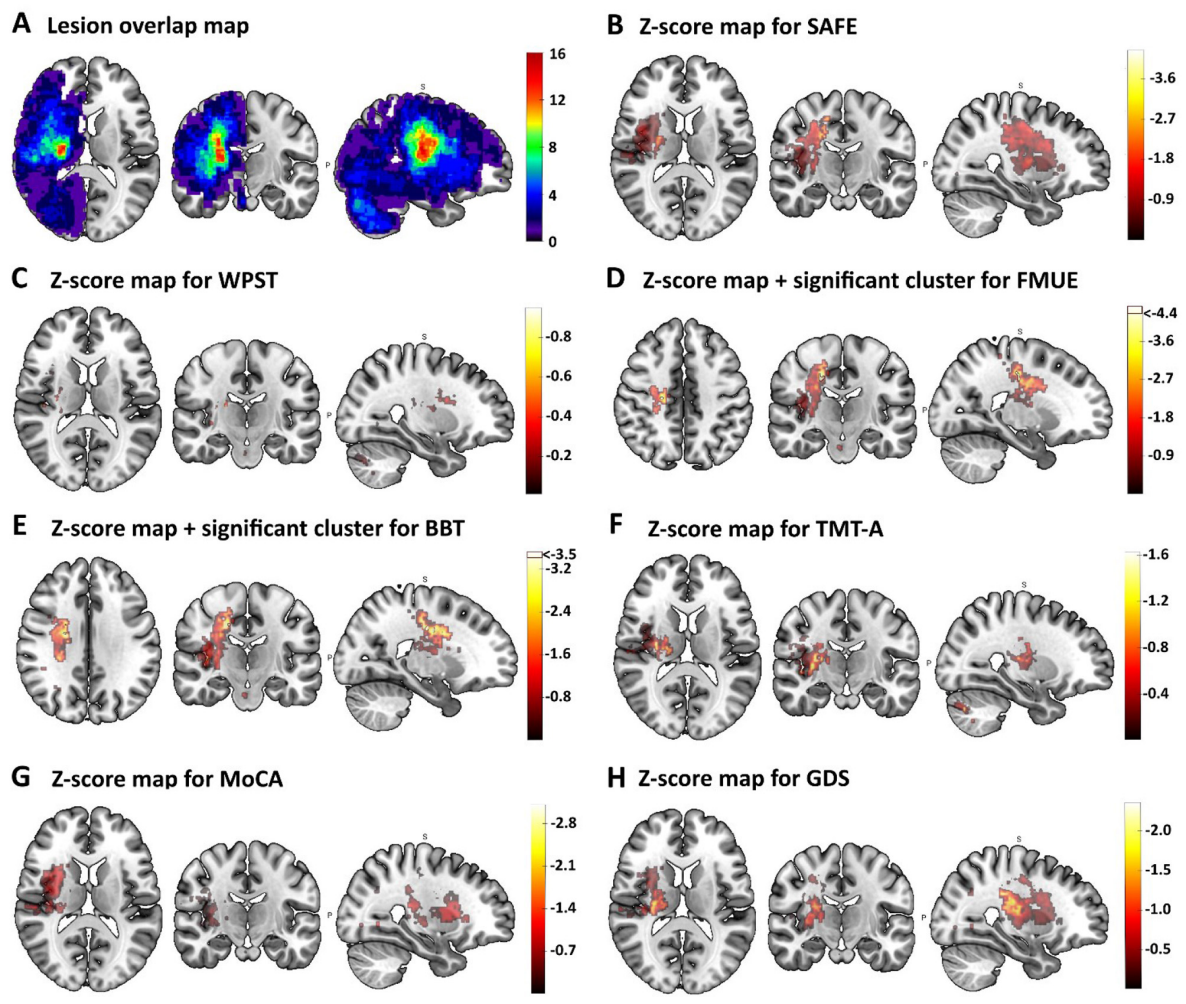
**(A)** Illustrates the lesion overlap masks for all 55 subjects. Lesions in left brain are flipped along the sagittal plane to the right side to gain statistical power when running the VLSM. **(B–H)** Show the Z-score maps and significant cluster (black outlined cluster) for the seven behavioral outcomes based on VLSM. **(D, E)** Show the significant lesion cluster overlaying regions of the corticospinal tract that were associated with lower FMUE (23 voxel, *z* = −4.4, *p* < 0.05), and lower performance in BBT (12 voxel, *z* = −3.5, *p* < 0.05).

A total of 60 patients with subacute stroke were enrolled from January 2022 to December 2024. Lesion-related neuroimaging measures were available for 55 of these; in five subjects, the stroke lesion could not be identified or processed due to low resolution of the neuroimages or timing of the scan. Participants had mean age 70.36 and were 42% females, with other characteristics appearing in Table 1. The mean lesion volume was 29.45 ml (range 0.096–354.6 ml). An overlap map of the 55 stroke lesions, all projected to the right hemisphere for the VLSM, appears in Figure 1A. Injury to the CST emanating from M1 was present in 70.7%; CST from PMd, 60%; and CST from PMv, in 65.5% of participants. Injury to the TST occurred in 56.4% of patients. The mean and standard deviations of network disconnections as measured by ChaCo scores are presented in Table 2, with the most extensive disconnection found between VN-SMN, VN-VAN, and SMN-FPN.

**Table 1:** Participant characteristics.

**Characteristic**	**Descriptive statistics**	***n***
Age in years, mean ± SD	70.36 ± 15.36	55
Sex, female/male, *n* (%)	23/32 (42/58)	55
Paretic body side, left/right in *n* (%)	31/24 (56/44)	55
Time after stroke, mean ± SD	10.2 ± 4.8	55
SAFE score, median (Q1–Q3)	8 (8–10)	55
Wrist proprioception average error, mean ± SD	17.87 ± 10.75	52
FMUE, median (Q1–Q3)	57 (40–62)	53
BBT, mean ± SD	24.17 ± 15.17	53
Montreal Cognitive Assessment, median (Q1–Q3)	21 (18–24)	54
Trail making test-A time, mean ± SD	68.02 ± 34.51	54
Geriatric Depression Scale, median (Q1–Q3)	3 (2–6)	55
Lesion volume in ml, mean ± SD	29.45 ± 57.64	55
Percent M1-CST tract injury, mean ± SD	39.0 ± 37.0	55
Percent PMd-CST tract injury, mean ± SD	36.6 ± 37.6	55
Percent PMv-CST tract injury, mean ± SD	25.5 ± 31.8	55
Percent TST injury, mean ± SD	39.6 ± 39.8	55

BBT, Box and Blocks Test; CST, corticospinal tract; FMUE, Fugl-Meyer Assessment of the Upper Extremity, M1, primary motor cortex, PMd, dorsal premotor cortex, PMv, ventral premotor cortex; Q1–Q3, first quartile to third quartile, SAFE, Shoulder Abduction and Finger Extension strength; SD, standard deviation; TST, thalamocortical sensory tract.

**Table 2:** Stroke lesion-related neural network disconnections.

**Lesion-related network disconnection**	**ChaCo score**
VN-VN disconnection	0.034 ± 0.103
VN-SMN disconnection	0.133 ± 0.188
VN-DAN disconnection	0.055 ± 0.119
VN-VAN disconnection	0.115 ± 0.182
VN-LMN disconnection	0.052 ± 0.145
VN-FPN disconnection	0.073 ± 0.132
VN-DMN disconnection	0.059 ± 0.128
SMN-SMN disconnection	0.059 ± 0.105
SMN-DAN disconnection	0.086 ± 0.132
SMN-VAN disconnection	0.081 ± 0.125
SMN-LMN disconnection	0.073 ± 0.116
SMN-FPN disconnection	0.104 ± 0.157
SMN-DMN disconnection	0.062 ± 0.093
DAN-DAN disconnection	0.040 ± 0.074
DAN-VAN disconnection	0.080 ± 0.122
DAN-LMN disconnection	0.043 ± 0.109
DAN-FPN disconnection	0.062 ± 0.105
DAN-DMN disconnection	0.052 ± 0.085
VAN-VAN disconnection	0.050 ± 0.099
VAN-LMN disconnection	0.041 ± 0.091
VAN-FPN disconnection	0.064 ± 0.118
VAN-DMN disconnection	0.046 ± 0.084
LMN-LMN disconnection	0.008 ± 0.028
LMN-FPN disconnection	0.035 ± 0.084
LMN-DMN disconnection	0.017 ± 0.038
FPN-FPN disconnection	0.042 ± 0.093
FPN-DMN disconnection	0.041 ± 0.077
DMN-DMN disconnection	0.018 ± 0.036

Results are mean ± SD; *n* = 60 for all analyses. ChaCo, change in connectivity; DAN, dorsal attention network; DMN, default mode network; FPN, frontoparietal network; LMN, limbic network; SD, standard deviation; SMN, sensorimotor network; VAN, ventral attention network; VN, visual network.

### 3.1 Association between behavior and direct lesion injury with VLSM

VLSM was included to test for associations between behavior and injury anatomy, and to assesses the specificity of such relationships. Figures 1B–H show the z-score maps for all seven of the behavioral outcomes assessed, with lesion clusters highlighted when significantly associated with an outcome. For UE sensorimotor outcomes, VLSM found consistently significant lesion clusters that overlay with different segments of the CST at the dorsal portion as well as the posterior limb of the internal capsule that were associated with poorer motor status. These CST lesion-clusters were associated with, lower FMUE scores (23 voxels, *z* = −4.53, *p* < 0.05, Figure 1D), and lower BBT scores (12 voxels, *z* = −3.57, *p* < 0.05, Figure 1E), but not with TMT-A (Figure 1F), MoCA (Figure 1G) or GDS scores (Figure 1H), indicating specificity in terms of which behaviors are related to lesions in this brain area. The VLSM for error in WPST produced a lesion cluster overlaying the ventral posterior lateral thalamus, but this did not reach significance (Figure 1C). VLSM for cognition and mood did not result in significant lesion clusters, with non-significant clusters overlaying areas such as the anterior insula for MoCA (Figure 1G), and GDS (Figure 1H).

### 3.2 Association between behavior and indirect regional and network disconnection measures

**Figure 2:**
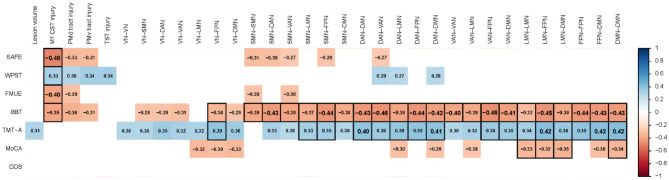
Statistically significant Spearman rank correlations (r) between behavior and lesion characteristics are shown in the corresponding color as defined in the color legend, when significant in uncorrected analyses; black outlined cells indicate correlations that remained significant after false discovery rate correction (FDR-corrected); correlation coefficients r ≥ 0.4 are displayed in a larger font size. BBT, Box and Blocks Test; DAN, dorsal attention network; DMN, default mode network; FMUE, Fugl-Meyer Assessment of the Upper Extremity; FPN, frontoparietal network; GDS, Geriatric Depression Scale; LMN, limbic network; M1CST, primary motor corticospinal tract; MoCA, Montreal Cognitive Assessment; PMd tract, dorsal premotor tract; PMv tract, ventral premotor tract; SAFE, Shoulder Abduction and Finger Extension strength; SMN, sensorimotor network; TMT-A time, Trail Making Test-A time; TST, thalamocortical sensory tract; VAN, ventral attention (salience) network; VN, visual network; WPST, Wrist Proprioception Sense Test.

The relationships that the seven behavioral measures (elementary sensorimotor behaviors, complex motor, and cognitive, behaviors, cognition, and mood) have with imaging measures of regional injury, and network disconnections are presented in Figure 2, where Spearman rank correlations are presented when significant in uncorrected analyses; black outlined cells indicate correlations that remained significant after false discovery rate correction (FDR-corrected). The strongest correlation after FDR correction was found between poorer SAFE score and greater M1-CST injury (*r* = −0.48, *p* = 0.001). In addition, greater M1-CST injury correlated with poorer FMUE (*r* = −0.40, *p* = 0.009), BBT (*r* = −0.39, *p* = 0.009), and poorer wrist proprioception (higher WPST error; *r* = 0.33, *p* = 0.03). Note that in this population lesion volume was not significantly (FDR-corrected) related to any of the seven behavioral measures.

Furthermore, scores on elementary sensorimotor behaviors showed a limited non-significant relationship with regional injury measures (PMd tract injury, PMv tract injury, TST injury) and network disconnections with SMN and DAN.

**Figure 3:**
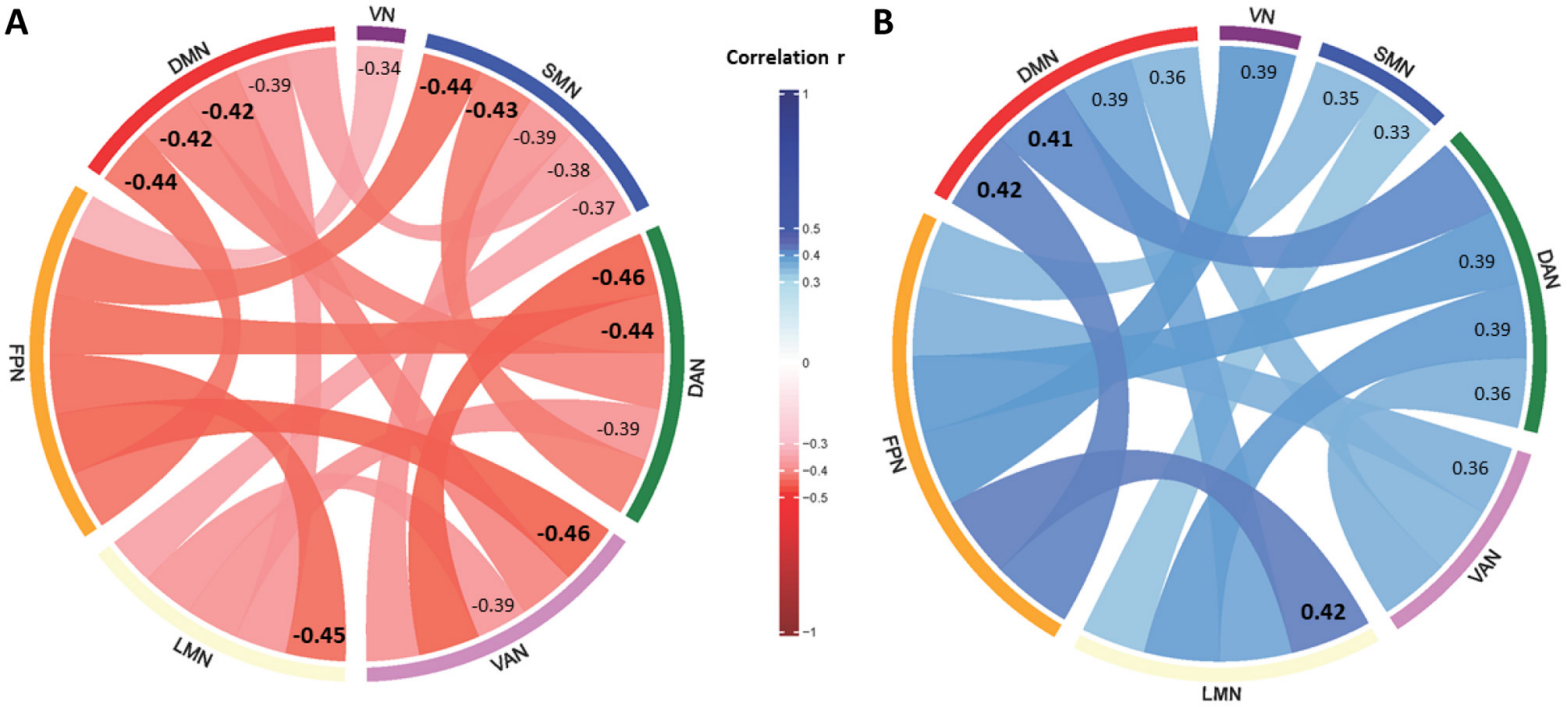
Significantly correlated network disconnections are presented in colored chords as defined in the color legend in the center. Correlation coefficients are additionally displayed for each disconnection chord and emphasized by a bold and increased font size for r ≥ 0.4. Figure 3A shows network disconnections associated with performance in the Box and Blocks Test and Figure 3B shows network disconnections associated with times needed to complete the Trail Making Test-A. DAN, dorsal attention network; DMN, default mode network; FPN, frontoparietal network; LMN, limbic network; SMN, sensorimotor network; VAN, ventral attention network; VN, visual network.

On the other hand, scores on complex motor and cognitive tasks were significantly (FDR-corrected) and extensively related to widespread network disconnections. Poorer performance on the BBT was significantly related to greater disconnection, for all networks and nearly all network pairs except of the VN (Figures 2, 3A). In particular, all network disconnections to-and-from the SMN, DAN, VAN and FPN were significantly and moderately related to poorer BBT scores, with r values ranging from−0.32 (*p* = 0.003) for BBT vs. LMN-LMN ChaCo disconnection scores, to−0.46 (*p* = 0.009) for BBT vs. ChaCo disconnection scores for both FPN-VAN and DAN-VAN. Similarly, longer time needed to complete the TMT-A correlated significantly with greater disconnections to-and-from all networks and nearly all network pairs (Figures 2, 3B). Amongst the strongest significant correlations with longer TMT-A time was greater ChaCo disconnection scores for LMN-FPN (*r* = 0.42, *p* = 0.009) as well as and DMN-FPN (*r* = 0.42, *p* = 0.009).

### 3.3 Modeling behavior with regional injury and network disconnection

Multivariable regression models were computed to test the explanatory strength that neuroimaging measures have in explaining the different baseline behavioral deficits. The most important variables for each outcome were chosen based on LASSO for each model. All models are reported in Table 3. Several models found that measures of disconnection explained outcome measures. Variance in FMUE was significantly explained by VN-VAN disconnections (β-estimate = 41.27 points, *p* = 0.0142), with days post-stroke also surviving as significant (*R*^2^ = 0.24, *R*^2^ 4-fold cross validation = 0.02). Variance in TMT-A was significantly explained by disconnections between VN and FPN (β-estimate = 79.80 s, *p* = 0.0347), with age retained as a significant explanatory variable, with an R^2^ of 0.24 that decreased to 0.06 under 4-fold cross-validation. The model for explaining MoCA scores included VAN-VAN disconnections (β-estimate = −18.77, *p* = 0.0347), with age and paretic side surviving as explanatory variables (*R*^2^ = 0.24, *R*^2^ 4-fold cross validation = 0.02). The model for explaining SAFE scores at baseline suggests M1CST injury as a significant variable that accounts for a loss of on average 2.19 points (*p* = 0.034) in SAFE scores besides controlling for days post-stroke (β-estimate = −0.26, *p* = 0.002). The model had an R^2^ of 0.24, dropping to 0.16 under 4-fold cross-validation, indicating that its predictive ability diminished when tested on unseen data and reflecting modest generalizability. The model for explaining BBT scores included sex as the only significant explanatory variable (*R*^2^ = 0.21, *R*^2^ 4-fold cross validation = 0.05). The models for explaining WPST or GDS had no significant explanatory variables.

**Table 3:** Multivariable regression models.

**Outcome behavior**	**Explanatory variable**	**Estimate**	**Std.Error**	***t*-value**	**Pr(>|*t*|)**	
SAFE	(Intercept)	10.98243	0.90183	12.178	<**0.001**	^***^
	M1CST injury	−2.19428	1.00771	−2.177	**0.034**	^*^
	Days post-stroke	−0.25846	0.07823	−3.304	**0.00173**	^**^
	R^2^ (R^2^ 4-fold cv)	0.2369 (0.1599)	2.7		**0.0003316**	^***^
WPST	(Intercept)	9.1079	8.5249	1.068	0.291	
	TST injury	5.17816	3.88664	1.332	0.189	
	SMN-FPN	14.73885	15.21211	0.969	0.338	
	DAN-DMN	43.84388	29.01359	1.511	0.138	
	LMN-LMN	−166.29965	111.88059	−1.486	0.144	
	Age	0.08621	0.10156	0.849	0.4	
	Days post-stroke	−0.20102	0.31681	−0.635	0.529	
	R^2^ (R^2^ 4-fold cv)	0.269 (0.0122)	9.192		**0.002198**	^**^
FMUE	(Intercept)	68.5866	7.0147	9.778	**8.28E**−**13**	^***^
	M1CST injury	−15.6065	9.8797	−1.58	0.121	
	**VN-VAN**	41.273	16.1937	2.549	0.0142	^*^
	SMN-FPN	−5.8734	29.4438	−0.199	0.8428	
	DMN-DMN	−134.2636	111.8639	−1.2	0.2362	
	Sex (Male)	−7.2288	4.9211	−1.469	0.1487	
	Days post-stroke	−1.1235	0.5179	−2.169	**0.0353**	^*^
	R^2^ (R^2^ 4-fold cv)	0.2402 (0.020)	16.64		**0.004109**	^**^
BBT	(Intercept)	57.7755	13.3974	4.312	**8.45E**−**05**	^***^
	M1CST injury	−7.2605	7.7316	−0.939	0.3526	
	SMN-FPN	−12.6033	38.3139	−0.329	0.7437	
	DAN-VAN	−10.29	48.7134	−0.211	0.8336	
	Age	−0.2063	0.145	−1.423	0.1614	
	Sex (Male)	−8.9242	3.9458	−2.262	**0.0285**	^*^
	Days post-stroke	−0.888	0.4678	−1.898	0.0639	
	R^2^ (R^2^ 4-fold cv)	0.2128 (0.053)	13.46		**0.008115**	^**^
TMT-A	(Intercept)	14.0292	21.3826	0.656	0.5148	
	**VN-FPN**	79.7989	36.7485	2.171	**0.0347**	^*^
	SMN-FPN	35.7298	29.5789	1.208	0.2327	
	Age	0.6277	0.302	2.079	**0.0428**	^*^
	R^2^ (R^2^ 4-fold cv)	0.2416 (0.059)	30.05		**0.0007403**	^***^
MoCA	(Intercept)	32.5859	3.2195	10.121	**4.60E**−**13**	^***^
	PMd tract injury	0.7957	3.7173	0.214	0.8315	
	PMv tract injury	3.1692	4.2081	0.753	0.4554	
	TST injury	2.1965	2.4816	0.885	0.3809	
	VN-LMN	−6.2858	8.2373	−0.763	0.4495	
	DAN-LMN	−6.4416	11.3447	−0.568	0.5731	
	VAN-VAN	−18.7735	8.6012	−2.183	**0.0344**	^*^
	Age	−0.147	0.0421	−3.492	**0.0011**	^**^
	Sex (Male)	−0.8929	1.2563	−0.711	0.481	
	Paretic side (Right)	−3.8616	1.2513	−3.086	**0.0035**	^**^
	R^2^ (R^2^ 4-fold cv)	0.3365 (0.06)	4.15		**0.0008886**	^***^

Multivariable regression models are presented for each behavioral outcome. Significant results and neuroimaging predictors are highlighted in bold. Model performance is evaluated by R^2^ and R^2^ under 4-fold cross validation and 10 runs. BBT, Box and Blocks Test; CST, corticospinal tract; cv, cross validation; FMUE, Fugl-Meyer Assessment of the Upper Extremity; M1, primary motor cortex; DAN, dorsal attention network; DMN, default mode network; FPN, frontoparietal network; LMN, limbic network; SAFE, Shoulder Abduction and Finger Extension strength; SMN, sensorimotor network; VAN, ventral attention network; VN, visual network. ^*^P < 0.05, ^**^P < 0.01, ^***^P < 0.001.

## 4 Discussion

The present study examined associations that behavioral measures have with brain regional injury and structural network disconnection in patients with subacute ischemic stroke. Consistent with the existing evidence, (Lin et al., 2019; Feng et al., 2015; Yoo et al., 2023; Boyd et al., 2017) measures of elementary UE sensorimotor behavior, such as strength and impairment, were significantly related to extent of CST injury. On the other hand, measures of complex motor and cognitive behaviors (BBT and TMT-A) were explained by the extent of structural disconnections between neural networks. In the current patient cohort, lesion volume did not significantly explain any of the behaviors (after FDR correction). Exploratory LASSO regression examining all clinical and neuroimaging measures found complex patterns of structural network disconnection across behaviors. Taken together, the results support the idea that the integrity of distributed connections across multiple large-scale brain networks is more important to complex motor and cognitive behaviors as compared to elementary sensorimotor behaviors.

For elementary sensorimotor behaviors, injury to specific white matter tracts was associated with deficits in corresponding behaviors. For example, in VLSM analyses, lower FMUE and BBT scores were significantly associated with lesion clusters in the posterior limb of the internal capsule. These findings were replicated when testing for associations between behavioral outcomes, and the percentage of M1CST injury. These results are consistent with a growing body of literature that identifies the CST as a critical neuroimaging biomarker for predicting motor recovery following stroke (Lin et al., 2019; Feng et al., 2015), and such measures of CST lesion load have been found to have high sensitivity, specificity, and positive predictive value compared to other neuroimaging or neurophysiological measures (Feng et al., 2015). These convergent observations underscore the utility of CST injury as a reliable indicator of motor status post-stroke, here validated in a cohort of patients who were an average of 10 days after stroke onset. No such associations were found between CST injury and cognitive or mood measures, underscoring that such biomarkers of sensorimotor system injury are specific to UE sensorimotor status.

On the other hand, complex motor and cognitive deficits were related to structural disconnection between brain networks. Deficits in BBT and TMT-A were significantly related to many network disconnections (Figures 2, 3). Some deficits in complex motor and cognitive behaviors were also related to injury to sensorimotor structures such as CST. The BBT assesses gross hand dexterity across an extended period of time and the TMT-A depends on psychomotor speed and visuospatial attention. Both tests are time-constrained and require the sequential execution of repetitive activities (e.g., grasping and releasing blocks, connecting numbers by lines). Current findings indicate that successful performance on these two tasks depends on the integrity of structural connectivity between multiple distributed brain networks related to attention, visuospatial and motor processing speed, planning, as well as sensorimotor function. For individuals' post-stroke, such tasks often require identifying effective compensatory strategies within the constraints of their motor and/or cognitive status. Moreover, although these tasks do not constitute dual-task paradigms in the classical sense, their demands may better approximate the complexity of everyday activities as compared to tests of elementary sensorimotor behavior.

Deficits in performing complex motor and cognitive tasks thus correspond to disconnection syndromes (Geschwind, 1965a,b) given that they correspond to substantial structural disconnectivity in brain networks. Some differences were seen between structural disconnection patterns, with BBT performance more related to reduced network connectivity between SMN, DAN, VAN, and FPN; and with TMT-A performance more related to reduced network connectivity between DMN, FPN, and LMN. Clinically, it is evident that some individuals can adapt to achieve task goals (through wrist positioning, use of momentum, head movements, or other compensations) while others appear less able to formulate or execute such strategies. For a given patient, this emphasizes the importance of considering the cognitive demands for sensorimotor tasks that they may find difficult. Overall, such results emphasize the importance of cognitive-motor interactions in post-stroke functional performance.

The large number and overlap of network disconnections (e.g., between FPN, DAN, VAN, LMN and DMN) associated with BBT and TMT-A performance are well supported by existing evidence (Lin et al., 2021; Varjacic et al., 2018). Brain areas for which structural integrity has been associated with BBT performance post-stroke include the dorsal anterior insula, which is part of the salience network (VAN) and is important for complex cognitive function (Lin et al., 2021). Reviewing studies that examined neural injury or functional connectivity in healthy subjects, Varjacic et al. found that large-scale networks in prefrontal and parietal cortices mediate TMT performance, consistent with current findings (Figures 2, 3) (Varjacic et al., 2018). Current results support the suggestion that integrity of structural connectivity across large scale neural networks may be a useful biomarker of TMT-A performance that could be examined as a therapeutic target for non-invasive brain stimulation (Dallasta and Marsh, 2024) and here suggest that this remains true in the early subacute phase post-stroke.

The importance of intact connectivity across large neural networks as a means to understand inter-subject differences in stroke-related deficits is supported by a growing body of research. For example, the DMN is inactive during task performance, in contrast with activity in DAN and VAN, and is active during certain cognitive processes such as self-reflection or mind wandering (Menon, 2023). Reduced DMN connectivity and activity after stroke has been linked with both cognitive and motor dysfunction (Tuladhar et al., 2013; Dahms et al., 2024; Lariviere et al., 2018). Wu et al. (2020) reported that synchrony between DMN, and SMN, specifically between the posterior cingulate cortex and the supplementary motor area, and between posterior cingulate cortex and M1, correlated with improvements in arm motor status (FMUE) over the course of rehabilitation and follow up.

Understanding the impact that structural network disconnection has on complex behavioral tasks is critical for a comprehensive understanding of post-stroke recovery, particularly concerning a patient's capacity for activities of daily living. The FPN, for example is important for grasping and prospective action judgments (Geers et al., 2021; Mutha et al., 2014) and showed increased activation after stroke that correlates with motor recovery especially when the CST was injured (Olafson et al., 2022). In this study, most of the FPN disconnections were consistently associated with poorer gross manual dexterity as tested with the BBT and visuospatial processing speed as assessed with the TMT-A. Similarly, the successful execution of complex goal-oriented tasks, such as those assessed by the BBT and TMT-A, critically relies on the coordination of multiple neural networks. The DAN provides top-down control for sustained visuospatial attention, movement planning, and suppression of irrelevant information, while the VAN, or salience network, facilitates the dynamic reorienting of attention, handling of unexpected events and integrates critical sensory feedback, such as the tactile sensation of grasping a block or the sound of a block dropping. The models presented herein suggest that disconnections between VN and VAN might be associated with better FMUE score, while disconnections between VN and FPN explain longer times needed in the TMT-A, and VAN-VAN disconnections were related with worse MoCA scores. Although the current study is only cross-sectional, the results support the value of considering more distributed neural network measures in stroke research, where direct lesion correlates face limitations. A recent study reported significant associations between FMUE changes and DAN functional connectivity during fMRI that were modulated by tDCS, (Salazar et al., 2024) providing evidence of how activity within brain networks can be modulated, and how this is related to behavioral changes. Identifying relevant brain networks or functional nodes that are related to certain behavioral phenotypes (and their recovery over time) may suggest new treatment targets for which the aim is to favorably modify connectivity within, and between networks. Such network connectivity measures might additionally inform stroke clinicians and researchers regarding the basis for specific behavioral deficits, potential for recovery over time, as well as facilitators and barriers to benefit from certain therapies. However, longitudinal and interventional studies are needed to confirm, and validate these findings, which could then inform the development of personalized, optimized rehabilitation therapies.

The current study examined structural brain injury (lesion overlap with canonical brain structures plus VLSM) and structural network disconnections, revealing that structural network disconnections best explained performance on complex motor and cognitive tasks for which performance requires both substantial motor execution and cognitive activity. Thus, it is important to consider the indirect effects of the neural injury on network connectivity when aiming to understand and treat deficits in more complex behaviors that imply a higher ecological validity to real-world activities. Future research may expand on the association between structural disconnections and changes in behavioral deficits after stroke as well as on investigating the association between network disconnections and task complexity by considering experimental designs that allow the gradual comparison between simple- and dual-task constructs.

Several strengths and limitations must be considered when interpreting current findings. The availability of neuroimaging measures such as regional injury measures and structural disconnection measures that are derived from acute clinical stroke imaging as well as a comprehensive battery of behavioral outcome measures, is a strength. The behavioral scales were assessed on average 10 days post-stroke, which can be considered a reasonable time frame for correlations with acute neuroimaging measures. Hence future studies should additionally look at association between neural correlates and behavior with respect to changes in deficits over time and with respect to different treatment effects. Despite enrollment of patients meeting entry criteria, one key limitation of this study is the high overlap of stroke lesions in deep subcortical regions with limited overlap in frontal and occipital cortical regions, which reduces power to probe the latter areas for injury-behavior relationships and so might have influenced the neutral results for cognition and mood. Note that the current subcortical distribution of infarct locations is consistent with prior research (Corbetta et al., 2015; Schwarz et al., 2025). Furthermore, the fact that many higher order cognitive deficits are not specifically related to a certain brain region and rather to more indirect network disconnections supports the utility of our methodological approach even with the unbalanced distribution of lesion locations. Finally, most associations between behavioral deficits and neural network disconnection were moderate in strength, as the deficit profile of enrolled patients affected the statistical power to examine certain relationships. Similarly, the strength and robustness of the LASSO regression models were low to modest, warranting caution in interpreting the findings. Validating the results in independent study cohorts with larger sample sizes and longitudinally over time will be important next steps.

In sum, this study confirmed the relevance of injury to specific sensorimotor brain areas, such as CST, for understanding elementary sensorimotor deficits after stroke. The results also indicate that more complex motor and cognitive behaviors rely on intact connections between multiple brain networks. Together, the results emphasize the importance of understanding stroke as a network disorder. Such findings may help explain the variability and complexity in stroke-related deficits and in the long term may foster novel approaches to examining patients after stroke toward the goal of personalized, optimized rehabilitation therapies.

## Data Availability

The datasets presented in this article are not publicly available and will be shared upon request, without undue reservation. Requests to access should be directed to sccramer@mednet.ucla.edu.
